# Seroprevalence of anti-SARS-CoV-2 in patients with hepatitis B and C: a pre-vaccination study

**DOI:** 10.1016/j.bjid.2024.103740

**Published:** 2024-04-23

**Authors:** Lucas Lima da Silva, Lia Laura Lewis-Ximenez, Mônica de Avelar Figueiredo Mafra Magalhães, Vanessa Salete de Paula, Livia Melo Villar

**Affiliations:** a Fundação Oswaldo Cruz (FIOCRUZ), Instituto Oswaldo Cruz (IOC), Laboratório de Hepatites Virais, Rio de Janeiro, RJ, Brazil; b Fundação Oswaldo Cruz (FIOCRUZ), Instituto de Comunicação e Informação Científica e Tecnológica em Saúde, Rio de Janeiro, RJ, Brazil; c Fundação Oswaldo Cruz (FIOCRUZ), Instituto Oswaldo Cruz (IOC), Laboratório de Virologia Molecular, Rio de Janeiro, RJ, Brazil

**Keywords:** Antibodies, COVID-19, Hepatitis, Seroprevalence

## Abstract

The serological markers for the diagnosis of COVID-19 plays an important role in the epidemiological investigation of the pandemic. This study aims to assess the prevalence of anti-SARS-CoV-2 in hepatitis B and C patients in a pre-vaccination of COVID-19 period. Between March 2020 and January 2021, 199 serum samples from individuals with HBsAg/HBV DNA or anti-HCV/HCV RNA positivity were tested for antibodies (IgM and IgG) against SARS-CoV-2 using Electrochemiluminescent Immunoassay (ECLIA). Among these, 50.3 % (100/199) tested positive for hepatitis C virus infection and 49.7 % (99/199) for hepatitis B virus, confirmed through molecular and serological diagnosis. The anti-SARS-CoV-2 seroprevalence was 24.1 % (48/199) in this population, with 23.23 % (23/99) hepatitis B and 25 % (25/100) hepatitis C patients tested positive for anti-SARS-CoV-2. The higher seroprevalence of anti-SARS-CoV-2 (16.58 %, 33/199) was detected among those over-40 years of age and the month of November 2020 had the highest number of detections 9 % (18/199) with the majority living in impoverished and neglected neighborhoods in the city of Rio de Janeiro. We found a high prevalence of anti-SARS-CoV-2 in patients with viral hepatitis before COVID-19 vaccination. This demonstrates the high exposure of this population during the period of social isolation.

## Introduction

In March 2020, the World Health Organization established the spread of the Severe Acute Respiratory Syndrome Coronavirus-2 (SARS-CoV-2) as a pandemic.^1^ Brazil was strongly affected by the pandemic, especially when assessing the infection rate among target populations. In 2023, more than 37 million cases were identified with more than 700.000 deaths reported.^2^^,^^3^ Despite the high number of cases, Brazil has one of the lowest rates of diagnosis when compared to other countries.^4^

The seroprevalence of Coronavirus 2019 Disease (COVID-19) can be assessed by detecting anti-SARS-CoV-2 antibodies. The serological markers for the diagnosis of COVID-19 play an important role in the investigation of previous exposure, and thus evaluating the spread of the viral agent among communities, identifying asymptomatic cases, and follow-up upon seroconversion in relation to vaccination. Seroprevalence studies are essential to understand the dynamics of infection transmission.^3^

Vulnerable population groups were the most severely affected by the pandemic of COVID-19.^5^ Studies demonstrate seroprevalence among individuals from different population groups and carriers of different viral infections, nevertheless, there is a lack of information in the literature regarding individuals with chronic viral hepatitis. A seroprevalence of 21.6 % of anti-SARS CoV-2 was found among healthcare workers who directly attended COVID-19 cases before vaccination implementation in Brazil,^3^ while other studies in different countries report anti-SARS-CoV-2 IgG prevalence of 31 % in the first 12 months of the pandemic in people living with Human Immunodeficiency Virus.^6^

Despite available reports on the prevalence of these antibodies in specific populations, there is little information of SARS-CoV-2 in carriers of chronic liver conditions, such as hepatitis B and C patients. The purpose of this study was to assess the prevalence of antibodies against COVID-19 (anti-SARS-CoV-2) in patients with hepatitis B and C who were referred to the Brazilian Reference Laboratory for Viral Hepatitis before COVID-19 vaccination became available.

This was across-sectional study approved by the Oswaldo Cruz Foundation ethics committee (CAAE number 11,177,119.8.0000.5248). Between March 2020 and January 2021, 199 samples were selected from the Viral Hepatitis Laboratory biorepository collected from patients followed at their clinic. The inclusion criteria for the study were based on residual samples with positivity to HBsAg/HBV DNA or anti-HCV/HCV RNA, being followed at this clinic and age over 18. The exclusion criteria were absence of demographic data (age and gender).

Residual serum samples were tested for antibodies (IgM and IgG) against SARS-CoV-2 using the Elecsys® Anti-SARS-CoV-2 Cobas test (Roche diagnostics, Basel, Switzerland). The assay methodology is based on the use of a recombinant protein representing the Nucleocapsid (N) antigen of the virus in a double antigen sandwich assay format of the Electrochemiluminescent Immunoassay (ECLIA) which favors the detection of high affinity antibodies against SARS-CoV-2. This assay detects antibody titers, which have been shown to correlate positively with neutralizing antibodies in neutralization assays. The Cutoff Index (COI) determines that samples with a COI above or different from 1 are classified as reactive, according to the manufacturer's instructions.

The sociodemographic and epidemiological data regarding the patients (age, gender, address, and education level) were collected through an active search in physical medical records conducted by the ambulatory clinic's staff at the health care facility.

A total of 199 samples were retrieved for COVID-19 testing, 50.3 % (100/199) were infected with hepatitis C and 49.7 % (99/199) with hepatitis B. The mean age of this study population was 48 years (±15.03), with 56.3 % (112/199) being women (Table 1). Most samples had been collected in the second semester of 2020, between August and December (63 %, 126/199).

**Table 1:** Socio-epidemiological data of the individuals in the study (*n* = 199).

**Characteristics**	**Hepatitis B** **(*n*=99)**	**Hepatitis C** **(*n*=100)**	**Total** **(*n*=199)**
Mean age (years-old) ± SD	44 (±13.92)	52 (±14.89)	48 (±15.03)
Female (%)	51 (51.5 %)	61 (61 %)	112 (56.3 %)
Male (%)	48 (48.5 %)	39 (39 %)	87 (43.7 %)
Location (%)			
County of Rio de Janeiro	83 (84 %)	70 (70 %)	155 (78 %)
Other cities from Rio de Janeiro State	16 (16 %)	28 (28 %)	44 (22 %)
Anti-SARS-CoV-2 positive (%)	23 (23.23 %)	25 (25 %)	48 (24.1 %)

**Fig. 1:**
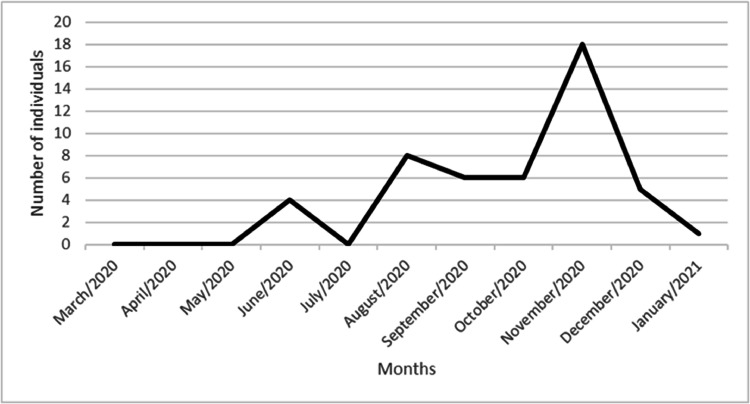
Number of anti-SARS-CoV-2 detections in relation to the pre-vaccination period evaluated in the study (March/2020‒January/2021).

Anti-SARS-CoV-2 positivity was detected in 24.1 % (48/199) samples. When stratified by viral hepatitis, 25 % (25/100) were infected with HCV and 23.23 % (23/99) with HBV. COVID-19 seroprevalence varied among the different age categories, 20‒40, 40‒60 and > 60, being respectively 8 % (16/199), 9.54 % (19/199) and 6.53 % (13/199). The COVID -19 seroprevalence by gender was 13 % (26/199) for women and 11 % (22/199) for men. When evaluating the educational level of only SARS-CoV-2 positive individuals, it was observed that 31.25 % (15/48) reported not having finished primary school, and the same number reported having completed high school. When evaluating the detection of anti-SARS-CoV-2 in relation to the period of time in which the patients had their blood collected (March/2020 and January/2021), the period between October to December of 2020, had the highest detection of anti-SARS-CoV-2. The highest number of detections being in November 2020, with, with 18 positive individuals 9 % (18/199), as depicted in Fig. 1.

**Fig. 2:**
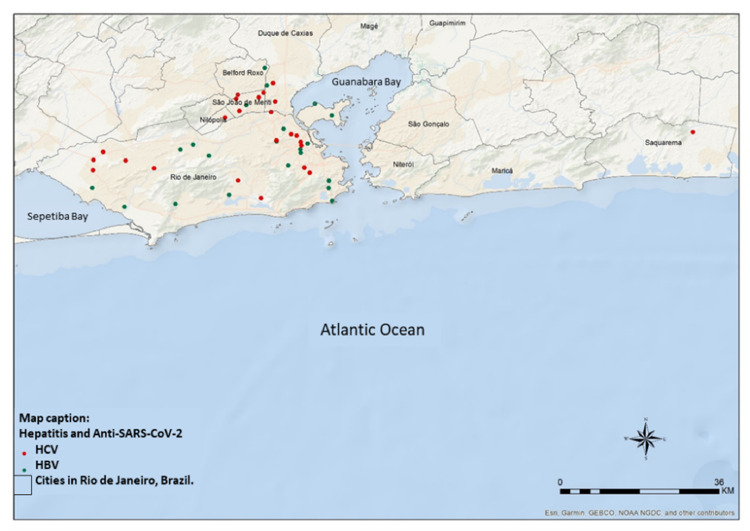
Geographical distribution of individuals in relation to their home address.

To demonstrate the geographical distribution of those who tested positive (*n* = 48) for anti-SARS-CoV-2 a map (Fig. 2) of the individuals was used based on available information from residence locations. Most cases concentrated in the county of Rio de Janeiro (73 %‒35/48), followed by other neighboring counties (27 %‒13/48).

There was a high prevalence of detection of antibodies against COVID-19 among patients with hepatitis, when compared to the general population. Although there is no reference data among people with chronic hepatitis to which the results of our study can be compared. In a study that evaluated the general population of several Brazilian cities, with more than 56.000 participants, prevalence rates of anti-SARS-CoV-2 in May 2020 of 1.6 % and 2.8 % in June 2020 were reported, with a considerable variation in detection between the regions of the country, and a higher level of prevalence in Rio de Janeiro state (5‒7 %), when compared to the states of the Brazilian southeast.^7^

When compared to studies evaluating populations with other co-infections, similar values were observed in the literature when compared to our work. A Brazilian study ^8^ detected anti-SARS-CoV-2 in 20.4 % among people living with HIV with no information on the prevalence of hepatitis among these individuals. This high prevalence may reveal that this population is exposed to SARS-CoV-2 even during a period of social isolation.

As for the age and gender of the individuals, most of the population was over 40 years, noting, a higher prevalence of anti-SARS-CoV-2 in this group and the highest seroprevalence was found in females. This prevalence of age from the fourth decade of life is consistent with the diagnosis and care of patients treated for viral hepatitis in health units.^9^ In a Brazilian study with more than 4.000 vulnerable individuals, the identification of female gender was higher among the population tested positive for Anti-SARS-CoV (59.9 %), in relation to age, the highest seroprevalence of anti-SARS-CoV-2 IgG was found in the 20‒29 age group (17.3 %), while in the 40‒49 age group the prevalence was 14.3 %.^10^ In a Thai study, carried out with more than 4.000 samples and over a timeframe similar to our study (May 14, 2020, and May 21, 2021), a similar average age was found when evaluating the prevalence of anti-SARS-CoV-2 among adults. However, there was a low prevalence of anti-SARS-CoV-2 detection, with 0.44 %.^11^ Data similar to the prevalence found in our study, are present in literature that evaluate younger populations. In a retrospective study carried out in Fortaleza, Brazil, with more than a thousand individuals, the seroprevalence was 25.3 % with IgG, IgM or IgG/IgM anti-SARS-CoV-2 among children and 29.2 % among adolescents.^12^

We found a higher detection of anti-SARS-CoV-2 among the samples collected in November 2020. The reduction in social isolation in the state of Rio de Janeiro, with the opening of shopping centers at reduced hours and the opening of outdoor spaces, was an important factor in the increase in cases during this period. Data provided by In Loco, a company specializing in geolocation, with 60 million mobile devices across the country and shared with the state government of Rio de Janeiro, shows that between April 12 and May 2, 2020, social isolation in the city of Rio was below 50 %.^13^ Epidemiological bulletins made available by other Brazilian states show an association between the drop in isolation in November 2020 and the increase in progressive positive cases for COVID-19 in the same period.^14^ Data from the national epidemiological report for November 2020 show that Rio de Janeiro was among the ten states with the highest number of new cases registered of COVID-19 and the highest mortality in the southeast.^15^

Among the geographical distribution of the individuals evaluated in our study, the highest concentration living in the city of Rio de Janeiro is notable. This is mostly due to the fact that the ambulatory clinic is located in this county and at least 72 % of the patients registered reside in the same county. The distribution between the neighborhoods in which these individuals live is vast and has concentration in city's poorest locations, this population has a low level of education, with 31 % (15/48) not having finished primary school and the same number having studied until high school. This is consistent with studies revealing the housing situation and vulnerability of populations living in impoverished slum communities in Brazil, with high seroprevalence of anti-SARS-CoV-2, reaching an overall prevalence of 49 %, which varied across different slums (from 3‒68 % to 4‒31 %) and the highest percentages of years of education in this population were in primary school (38 %) and in high school (37 %), reinforcing the vulnerability of these groups.^10^

This study found a high prevalence of Anti-SARS-CoV-2 in patients with viral hepatitis before COVID-19 vaccination. This shows the high exposure of this population at a time of social isolation. There are no studies in the literature that evaluate the history of exposure to SARS-CoV-2 in individuals infected with hepatitis B and C viruses, especially when evaluating the degree of vulnerability that this population may present in relation to their residence, age, gender and educational level, which reveals the authenticity and contributing to the literature by allowing other studies that investigate this specific population. These data can contribute to the planning of vaccination campaigns in key populations and the evaluation of the socio-epidemiological behavior of groups in relation to diseases.

## Conflicts of interest

The authors declare no conflicts of interest.
